# Prediction of chemical reaction yields with large-scale multi-view pre-training

**DOI:** 10.1186/s13321-024-00815-2

**Published:** 2024-02-25

**Authors:** Runhan Shi, Gufeng Yu, Xiaohong Huo, Yang Yang

**Affiliations:** 1 Department of Computer Science and Engineering, and Key Laboratory of Shanghai Education Commission for Intelligent Interaction and Cognitive Engineering, Shanghai Jiao Tong University, Shanghai, 200240 China; 2 Shanghai Key Laboratory for Molecular Engineering of Chiral Drugs, Frontiers Science Center for Transformative Molecules, School of Chemistry and Chemical Engineering, Shanghai Jiao Tong University, Shanghai, 200240 China

**Keywords:** Chemical reaction yield prediction, Self-supervised learning, Multi-view

## Abstract

Developing machine learning models with high generalization capability for predicting chemical reaction yields is of significant interest and importance. The efficacy of such models depends heavily on the representation of chemical reactions, which has commonly been learned from SMILES or graphs of molecules using deep neural networks. However, the progression of chemical reactions is inherently determined by the molecular 3D geometric properties, which have been recently highlighted as crucial features in accurately predicting molecular properties and chemical reactions. Additionally, large-scale pre-training has been shown to be essential in enhancing the generalization capability of complex deep learning models. Based on these considerations, we propose the Reaction Multi-View Pre-training (ReaMVP) framework, which leverages self-supervised learning techniques and a two-stage pre-training strategy to predict chemical reaction yields. By incorporating multi-view learning with 3D geometric information, ReaMVP achieves state-of-the-art performance on two benchmark datasets. Notably, the experimental results indicate that ReaMVP has a significant advantage in predicting out-of-sample data, suggesting an enhanced generalization ability to predict new reactions. Scientific Contribution: This study presents the ReaMVP framework, which improves the generalization capability of machine learning models for predicting chemical reaction yields. By integrating sequential and geometric views and leveraging self-supervised learning techniques with a two-stage pre-training strategy, ReaMVP achieves state-of-the-art performance on benchmark datasets. The framework demonstrates superior predictive ability for out-of-sample data and enhances the prediction of new reactions.

**Supplementary Information:**

The online version contains supplementary material available at 10.1186/s13321-024-00815-2.

## Introduction

The prediction of chemical reaction yields, which refer to the percentage of product formed in relation to the reactant consumed, is an important research topic in organic chemistry [1, 2]. In the field of organic synthesis, chemists often synthesize a target molecule through several or a dozen reaction steps [3]. Consequently, low-yield reactions in the intermediate steps can have a negative impact on the total yield of the synthesis route due to the cumulative effect of each step. The estimation of chemical reaction yields plays an important role in guiding synthetic chemists to choose appropriate molecular synthesis routes, particularly in the case of identifying highly active and selective catalysts efficiently. Traditionally, chemists depend on empirical predictions or specific wet experiments to determine yields, which require extensive domain knowledge and are both time-consuming and labor-intensive. Therefore, data-driven machine learning techniques are needed to provide an efficient alternative.

It remains a great challenge to accurately predict chemical reaction yields due to the complexity of reaction space and the diverse factors that influence chemical experiments [4, 5]. To develop machine learning-based approaches, it is crucial to establish effective methods for representing chemical reactions. Conventional studies typically rely on feature engineering to represent chemical reactions using fingerprints or descriptors. This involves creating customized descriptors based on domain expertise to capture molecular, atomic, vibrational, or physicochemical properties [6–12]. Some researchers derived descriptors from circular substructures present in the simplified molecular-input line-entry system (SMILES) [13] strings of the reactions [14]. Chemical reactions can be perceived as sequences or collections of molecules. Therefore, the traditional practice of concatenating molecular fingerprints or descriptors at the molecule level is commonly employed [15–18]. However, this concatenation approach is typically suitable only for reactions with a fixed number of molecules, posing limitations on their ability to generalize to downstream tasks.

In recent years, deep learning (DL) models have gained popularity and have led to significant advancements in the representation and prediction of chemical reactions. Some studies utilized SMILES as input and employed well-established models from the field of natural language processing (NLP) to encode the SMILES notations of chemical reactions into continuous vectors. These studies employed either pre-trained Transformer-based models [19–21] or Recurrent Neural Network (RNN) models [22, 23] on large-scale datasets, and fine-tuned their models [3, 24] with downstream tasks to capture task-specific representations of chemical reactions. Other studies incorporated molecular graph structures to represent chemical reactions. Recent approaches include adding [25] or concatenating [26] molecular features learned from graph neural networks, encoding the condensed graph of chemical reactions [27], and learning organic reactivity based on generalized reaction templates [28].

While the YieldGNN model [12] attempts to combine 2D chemical reaction graphs with 1D descriptors, most of the aforementioned studies have focused solely on a specific perspective of chemical information, namely, 1D sequences or 2D graphs. By contrast, multi-view learning methods for molecular representation learning [29–33] have achieved success by incorporating multiple views of molecules with different dimensional inputs.

However, due to the difficulty in extracting effective features from molecular geometry, only a few molecule prediction methods [30, 34–37] have leveraged 3D spatial structure information of molecules, which is critical for determining molecular properties and reaction outcomes. Although these studies have demonstrated the potential of 3D geometric information in providing comprehensive and complementary insights into chemical reactions, to further enhance the accuracy of reaction prediction, there is a need to explore more effective and efficient algorithms for handling the high dimensionality of 3D molecular structures.

**Fig. 1:**
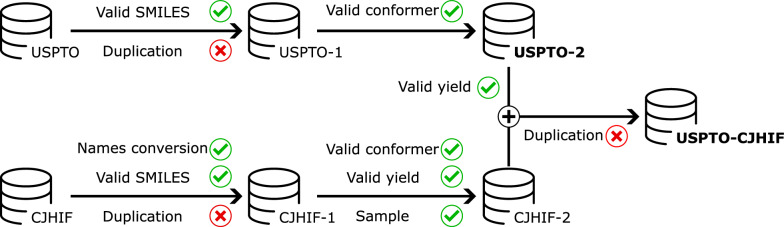
Overview of the data preparation process. We remove duplicate reactions and retain reactions with valid SMILES and conformers. We use USPTO-2 and USPTO-CJHIF for the first stage and the second stage of pre-training, respectively

In this study, we propose a large-scale **R**eaction **M**ulti-**V**iew **P**re-training framework, named ReaMVP, to represent chemical reactions and predict their yields. ReaMVP utilizes a two-stage approach that involves the pre-training of a sequence encoder and a conformer encoder. In the first stage, ReaMVP aims to capture the consistency of chemical reactions from different views via distribution alignment and contrastive learning. In the second stage, ReaMVP further enhances the representation of chemical reactions through supervised learning using reaction data with known yields. By incorporating this additional information, the model can improve its performance on downstream prediction tasks. The contributions of this work are summarized as follows:

1. We model chemical reactions through both sequential and geometric views, which enables the model to capture more abundant and comprehensive structural information. Additionally, we propose a simple yet effective approach to encode chemical reactions in the geometric view.
2. We propose a novel self-supervised pre-training method based on distribution alignment and contrastive learning using multiple views of chemical reactions, which has advantages in capturing the consistency between pairs of chemical reactions.
3. Leveraging the advantages of large-scale pre-training, the proposed ReaMVP provides high generalization capability in predicting chemical reaction yields. It outperforms the baseline models by a considerable margin under out-of-sample conditions where certain molecules are not seen in the training set.

## Materials and methods

### Data preparation

#### Pre-training datasets

Two large-scale datasets are utilized for the first and second stages of pre-training, respectively. Figure 1 illustrates the data preparation pipeline.

**Table 1:** Overview of the pre-training datasets

Dataset	# Reactions	# Training	# Validation	# Test	Task type
USPTO-2	1055411	949869	52771	52771	Self-supervised
USPTO-CJHIF	646631	581967	32332	32332	Supervised

The United States Patent and Trademark Office (USPTO) from 1976 to September 2016 [38] is a large database of reactions. The original dataset contains over 1.8 million chemical reactions stored in the form of SMILES arbitrary target specification (SMARTS) [39]. We remove duplicate records and invalid reactions that RDKit [40] fails to recognize, and then transform the remaining reactions into SMILES to obtain the dataset USPTO-1. Subsequently, we retain reactions in which RDKit is able to generate geometric information for all molecules to obtain the dataset USPTO-2, which is used in the first stage of pre-training.

For the second stage of pre-training, we select reactions from USPTO-2 that possess known and valid yields. However, the yield distribution in USPTO-2 is highly biased since it contains only a few reactions with low yields (the detailed distribution is present in Additional file 1: Figure S4), which potentially limits the model’s generalization ability. To address this issue, we augment USPTO-2 by adding more reactions with low yields from the Chemical Journals with High Impact Factor (CJHIF) [23] dataset to cover a wider range of values. The original CJHIF dataset includes over 3.2 million chemical reactions in the form of SMARTS extracted from chemistry journals by Chemical.AI [41], which only considers the reactants and products. However, additional compounds such as catalysts and solvents are represented using plain English names or abbreviations that cannot be directly recognized and processed by computers. To convert these names into RDKit-recognizable formats, we utilize both the open parser for systematic IUPAC nomenclature (OPSIN) [42] and the chemical identifier resolver (CIR) [43] to obtain their corresponding SMILES representations. Similar to the processing pipeline for USPTO, we further remove duplicate and invalid reactions and convert the remaining reactions into SMILES to obtain the dataset CJHIF-1. Subsequently, we retain reactions with known and valid yields and sample reactions whose yields are lower than $50\%$. The combination of chemical reactions with known yields from USPTO-2 and CJHIF forms the dataset USPTO-CJHIF, which is used in the second stage of pre-training.

Note that the conformer encoder necessitates the atom coordinates of each molecule. However, generating a dataset comprising millions of transition state reactions is exceedingly intricate and computationally demanding [44]. As a substitute, we employ molecular conformers to depict the geometric structures of reactions. Experimental determination of conformers involves resource-intensive physical and chemical experiments. Hence, we rely on computational chemistry, employing simulation software and algorithms to model molecule conformers. Specifically, we utilize the ETKDG algorithm [45] provided by RDKit with default parameters to compute one conformer for each molecule.

USPTO-2 is randomly divided into training, validation, and test sets in an 18:1:1 ratio. Similarly, USPTO-CJHIF is divided in a stratified way according to the yields of reactions in the same ratio as USPTO-2. Table 1 presents an overview of the datasets for pre-training.

**Table 2:** Overview of the downstream datasets

Dataset	Split type	# Training	# Test	Out-of-sample type
Buchwald-Hartwig	Test 1	3057	898	Ligand-based
(3955 reactions)	Test 2	3055	900	Ligand-based
	Test 3	3058	897	Ligand-based
	Test 4	3055	900	Ligand-based
	Plate 1	2880	1075	Ligand-based
	Plate 2	2515	1440	Lligand-based
	Plate 3	2515	1440	Ligand-based
	Plate 2 new	2515	1440	Ligand-based
	Halide Br	2636	1319	Reactant-based
	Halide Cl	2637	1318	Reactant-based
	Halide I	2637	1318	reactant-based
	Pyridyl	2372	1583	Reactant-based
	Nonpyridyl	1583	2372	Reactant-based
	random	2768	1187	None
Suzuki-Miyaura	Test 1	4320	1440	Ligand-based
(5760 reactions)	Test 2	4320	1440	Ligand-based
	Test 3	4320	1440	Ligand-based
	Test 4	4320	1440	Ligand-based
	Random	4032	1728	None

#### Downstream datasets

We fine-tune and assess ReaMVP on two benchmark datasets for the prediction of chemical reaction yields. Table 2 presents the data statistics.

It is noteworthy that, when predicting chemical reaction yields, chemists are often able to select appropriate reactants guided by the desired product. However, selecting influential precursors, such as additives and catalysts, that have a significant impact on yields poses a considerable challenge. This challenge necessitates the exploration of numerous unobserved alternative molecules, demanding a machine learning model with high generalization capability under out-of-sample conditions. Specifically, the model must be capable of accurately predicting yields of reactions that involve molecules not included in the training set.

The Buchwald-Hartwig dataset was released by Ahneman et al. [8]. They conducted high-throughput experiments (HTEs) with 1536-well plates on the class of Pd-catalyzed Buchwald-Hartwig C-N cross-coupling reactions. They experimented on the combinations of 15 aryl halides, four ligands, three bases, and 23 additives. A total of 3955 reactions were reported with their measured yields. Ahneman et al. and Sandfort et al. [15] split the dataset into eight representative training and test sets according to isoxazole additives as out-of-sample conditions. We further split the dataset based on reactants to construct five out-of-sample conditions (detailed split groups are shown in Additional file 1: Figure S1). We also apply the same random 70/30 split as reported in Sandfort et al. to get training and test sets.

The Suzuki-Miyaura dataset was released by Perera et al. [46]. They conducted high-throughput experiments on the class of Suzuki-Miyaura cross-coupling reactions. Discarding water (H_2_O), 15 couplings of electrophiles and nucleophiles across combinations of 12 ligands (with a blank one), eight bases (with a blank one), and four solvents were considered, resulting in measured yields for a total of 5760 reactions. To evaluate the model under out-of-sample conditions similar to the Buchwald-Hartwig dataset, we further split the Suzuki-Miyaura dataset into four representative training and test sets according to ligands (detailed split groups are shown in Additional file 1: Figure S3). We also apply the same random 70/30 split as reported in Philippe et al. [3] to get training and test sets.

### Problem formulation

Suppose that a chemical reaction contains a total of *n* molecules, including reactants, catalysts, products, and other relevant compounds. Each reaction is associated with a yield value, and the value of *n* may vary across different reactions. We denote each sample as $({\mathcal {M}}_s, {\mathcal {M}}_c, y)$, where ${\mathcal {M}}_s=\{S_1,\ldots ,S_n\}$ and ${\mathcal {M}}_c=\{C_1,\ldots ,C_n\}$ represent the set of *n* molecules in sequence format and conformer format, respectively, and *y* refers to the reaction yield. For the *i*-th molecule within a reaction, $S_i$ represents its molecular sequence, and $C_i=\{{\mathcal {V}}, {\mathcal {R}}\}$ represents its corresponding molecular conformer, where ${\mathcal {V}}=\{v_1,\dots ,v_m\}$ and ${\mathcal {R}}=\{r_1,\dots ,r_m\}$ denote the set of atoms and their spatial coordinates, respectively.

Given a reaction $({\mathcal {M}}_s^\prime , {\mathcal {M}}_c^\prime , y^\prime )$ where $y^\prime$ is unknown, the task of predicting reaction yields aims to find a mapping function that can be defined as follows:

1 $$\begin{aligned} \begin{aligned} y_p = \phi \left({{ \mathcal{M}}}_s^\prime , {{\mathcal {M}}}_c^\prime \right), \end{aligned} \end{aligned}$$

where $\phi (\cdot )$ represents the desired mapping function and $y_p$ denotes the predicted yield.

**Fig. 2:**
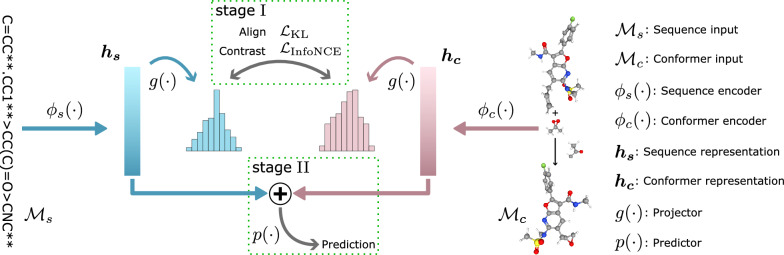
Overview of the model

### Model architecture

Figure 2 shows the basic structure of ReaMVP. As a generic self-supervised learning pipeline, the proposed model, ReaMVP, consists of two phases: pre-training and fine-tuning. The pre-training phase consists of two stages. In stage I, we map multiple views of reactions into the representation space via sequence (1D) and conformer (3D) encoders, respectively. The mapped representations are then projected into the alignment space where we conduct self-supervised pre-training. In stage II, we carry out supervised pre-training in the representation space.

#### Pre-training stage I

In the pre-training stage I, we aim to improve the representation of chemical reactions by conducting distribution alignment (Align) and contrastive learning (Contrast) using data collections that provide both sequential and geometric structures. To achieve this, ReaMVP utilizes the 1D sequence and the 3D conformer as complementary views for each reaction. We employ a sequence encoder $\phi _s(\cdot )$ and a conformer encoder $\phi _c(\cdot )$ to learn the representations of reactions. Subsequently, a projection head [47] $g(\cdot )$ (also called a projector) is applied to map the learned representations to the alignment space as follows:

2 $$\begin{aligned} \begin{aligned} \varvec{h_s}&= \phi _s({\mathcal {M}}_s),\ \varvec{h_c} = \phi _c({\mathcal {M}}_c),\\ \varvec{x_s}&= g(\varvec{h_s}),\ \varvec{x_c} = g(\varvec{h_c}), \end{aligned} \end{aligned}$$

where $\phi _s(\cdot )$ and $\phi _c(\cdot )$ represent the sequence encoder and the conformer encoder, $\varvec{h_s}$ and $\varvec{h_c}$ denote the learned representation of the corresponding encoders, and $\varvec{x_s}$ and $\varvec{x_c}$ are the output in the alignment space of the sequence encoder and the conformer encoder, respectively. Given a batch of *N* inputs $\varvec{X_s}=\{\varvec{x_{s_1}},\dots ,\varvec{x_{s_N}}\}$ and $\varvec{X_c}=\{\varvec{x_{c_1}},\dots ,\varvec{x_{c_N}}\}$, ReaMVP aims to align the representations of the two views for the same reaction, i.e., pushing $\varvec{x_{s_i}}$ and $\varvec{x_{c_i}}$ as close as possible. Hence we apply the Jeffreys divergence [48] to achieve this in a distribution-like format as follows:

3 $$\begin{aligned}{} & {} \begin{aligned} {\varvec{Z_s}}&= \text {LogSoftmax}(\varvec{X_s}),\\ {\varvec{Z_c}}&= \text {LogSoftmax}(\varvec{X_c}), \end{aligned} \end{aligned}$$

4 $$\begin{aligned}{} & {} \begin{aligned} {\mathcal {L}}_{\text {KL}}&= \frac{1}{2}{\mathbb {E}}_{p({\varvec{z_s}}, {\varvec{z_c}})} \left[ {D\left( {{\varvec{Z}_s}\parallel {\varvec{Z}_c}} \right) + D\left( {{{\varvec{Z}}_c}\parallel {{\varvec{Z}}_s}} \right)} \right] \\&= -\frac{1}{2N}\sum _{i=1}^{N}\sum _{j=1}^{d} \left( \varvec{z_{s_i}^j}\log \frac{\varvec{z_{c_i}^j}}{\varvec{z_{s_i}^j}} + \varvec{z_{c_i}^j}\log \frac{\varvec{z_{s_i}^j}}{\varvec{z_{c_i}^j}}\right) , \end{aligned} \end{aligned}$$

where *D* denotes the Kullback–Leibler divergence [49], *d* denotes the dimension of features, $\varvec{z_{s_i}}$ denotes the *i*-th input of $\varvec{Z_s}$, $\varvec{z_{s_i}^j}$ denotes the *j*-th logit of $\varvec{z_{s_i}}$, and $\varvec{z_{c_i}}$ and $\varvec{Z_{c_i}^j}$ are defined in a similar way. Furthermore, we want to separate the outputs from distinct views for different sample pairs as far as possible to enhance the representation ability of chemical reactions. Thus, we adopt contrastive learning based on InfoNCE [50] to maximize the mutual information between $\varvec{X_s}$ and $\varvec{X_c}$ as follows:

5 $$\mathcal{L}_\text{InfoNCE} = - \frac{1}{2}{\mathbb{E}}_{{p(\varvec{x_{s}} ,\varvec{x_{c}} )}} \left[ {\log \frac{{f(\varvec{x_{s}} ,\varvec{x_{c}} )}}{{f(\varvec{x_{s}} ,\varvec{x_{c}} ) + \sum f (\varvec{x_{{s^{\prime } }}} ,\varvec{x_{c}} )}}} \right. + \left. {\log \frac{{f(\varvec{x_{c}} ,\varvec{x_{s}} )}}{{f(\varvec{x_{c}} ,\varvec{x_{s}} ) + \sum f (\varvec{x_{{c^{\prime } }}} ,\varvec{x_{s}} )}}} \right] = - \frac{1}{{2N}}\sum\limits_{{i = 1}}^{N} {\left[ {\log \frac{{\exp \left( \varvec{x_{{s_{i} }} \cdot x_{{c_{i} }}} /{\tau } \right)}}{{\sum\limits_{{k = 1}}^{N} {\exp } \left( \varvec{x_{{s_{i} }} \cdot x_{{c_{k} }}} /{\tau } \right)}}} \right.} + \left. {\log \frac{{\exp \left( \varvec{x_{{c_{i} }} \cdot x_{{s_{i} }}} /{\tau } \right)}}{{\sum\limits_{{k = 1}}^{N} {\exp } \left( \varvec{x_{{c_{i} }} \cdot x_{{s_{k} }}} /{\tau } \right)}}} \right],$$

where *f*(*x*, *y*) equals $\exp (x\cdot y/\tau )$, $\tau$ denotes a hyper-parameter called temperature, $\varvec{x_{s^\prime }}$ and $\varvec{x_{c^\prime }}$ represent the sequence and conformer views of other reactions within the same batch, relative to positive pair $(\varvec{x_{s}}, \varvec{x_{c}})$, and $(\varvec{x_{s_i}}, \varvec{x_{c_k}})$ denotes the feature of the sequence encoder and the conformer encoder, respectively. The pair $(\varvec{x_{s_i}}, \varvec{x_{c_k}})$ comes from different reactions when *i* is not equal to *k* (negative pairs) and vice versa (positive pairs). To combine the distribution alignment and contrastive learning objectives, we formulate the overall loss function for the pre-training stage I as a combination of the distribution alignment loss in Equation (4) and the contrastive learning loss in Equation (5) as follows:

6 $$\begin{aligned} {\mathcal {L}}_{\text {I}} = {\mathcal {L}}_{\text {KL}} + \lambda \cdot {\mathcal {L}}_{\text {InfoNCE}}, \end{aligned}$$

where $\lambda$ denotes the weighting coefficient to balance the contributions of these two objectives.

#### Pre-training stage II

Focusing on the prediction of chemical reaction yields, we aim to improve the generalization capability of ReaMVP by leveraging supervised learning techniques (see “Both self-supervised and supervised pre-training enhance prediction performance” Section for details). Despite the inherent dissimilarity among various types of chemical reactions, we adopt the large-scale dataset USPTO-CJHIF for pre-training to capture the shared and common characteristics between chemical reactions and their corresponding yields.

In the pre-training stage II, we concatenate the learned representations from both the sequence encoder and the conformer encoder. The fused representations are then used for supervised pre-training, where a predictor is introduced to further improve performance on reaction yield prediction tasks. The loss function of the pre-training stage II is,

7 $$\begin{aligned} {\mathcal {L}}_{\text {II}} = \sum _{i=1}^N\left( y_i-p(\varvec{h_{s_i}}\oplus \varvec{h_{c_i}})\right) ^2, \end{aligned}$$

where $\oplus$ denotes the concatenation operation and $p(\cdot )$ denotes the predictor.

**Fig. 3:**
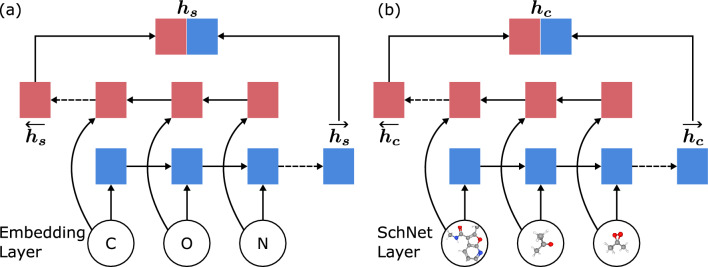
Structure of a one-layer bidirectional GRU as an example of the sequence encoder **a** and the conformer encoder **b**

#### Fine-tuning

The learned concatenated representations can be fixed as reaction descriptors or further trained during fine-tuning. In this study, we fine-tune the entire ReaMVP model along with the predictor for the problem of predicting reaction yields. We adopt the model on the Buchwald-Hartwig dataset and the Suzuki-Miyaura dataset, respectively.

#### Reaction sequence encoding

SMILES is a well-designed and widely-used sequence format to represent molecules, which has demonstrated its effectiveness in various chemistry-related tasks [3, 19–24]. Hence we adopt a multi-layer bidirectional gated recurrent unit (GRU) model [51] as the sequence encoder to process SMILES input as follows:

8 $$\varvec{h_s} = {\text{Bi - GRU }}\left( {\left\{ {\varvec{h_{{t_1}}}, \ldots ,\varvec{h_{{t_n}}}} \right\}} \right),{\text{ }}$$

where Bi-GRU denotes a multi-layer bidirectional gated recurrent unit model and $\varvec{h_{t_i}} (1\le i\le n)$ denotes the embedding of the *i*-th token. Figure 3a presents the basic structure of the sequence encoder.

Similar to natural language processing problems, tokenization is a key technology for the sequence encoder, and the level of granularity has a great impact on model performance [52]. Existing works for SMILES representation apply either a coarse-grained tokenization method [23] based on the statistical probability of characters or fields in the dataset, or a fine-grained tokenization method at the character level [3, 53, 54]. Considering the versatility of tokenization, we employ a fine-grained tokenization approach adapted from Xue et al. [54] based on the character level to ensure a high frequency of each token, except that the symbol “%” followed by two digits is segmented as one word. It is worth noting that aromatic elements are represented in lowercase when forming chemical bonds in the SMILES representation. Hence it is necessary to distinguish between cases such as “[*cs*]” and “*cs*”. The former is the representation of the lowercase element “*Cs*” in the aromatic bond, while the latter is the bond representation of the two elements “*c*” and “*s*”. In addition to the elements or other characters obtained from tokenization, we introduce five special characters to construct the pre-training task. The “[*PAD*]” symbol is used for aligning the length of input SMILES; the “[*CLS*]” symbol is used for recording classification information or indicating the start position of a SMILES string; the “[*SEP*]” symbol is used for separating reactants, catalysts, products, etc.; and the “[*UNK*]” symbol is used for representing characters that are either unknown or have a frequency less than ten. Consequently, the final corpus consists of 115 valid tokens (111 original tokens plus the additional 4 tokens).

#### Reaction conformer encoding

Although numerous studies have explored the use of 3D conformers to predict molecular properties [30, 34–37], there has been a notable dearth in the development of reaction representations and architectures that incorporate geometric information. In addition, models need to be rotational and translational invariant since conformers are generally described by atomic coordinates and are not fixed in the Cartesian or spherical coordinate systems. In light of these considerations, we propose a simple yet effective approach to capturing spatial features of reactions by utilizing sequential molecular conformers, as illustrated in Fig. 3b. The conformer encoder is composed of a SchNet [34] model that satisfies rotational and translational invariance to embed molecules and a multi-layer bidirectional GRU to aggregate molecular representations as follows:

9 $$\begin{aligned} \begin{aligned} \varvec{h_{m_i}} &= \text{SchNet}(C_i), \\ \varvec{h_c} &= {\text{Bi - GRU }}\left( {\left\{ {\varvec{h_{m_1}}, \ldots ,\varvec{h_{m_n}}} \right\}} \right),{\text{ }}\end{aligned} \end{aligned}$$

where $\varvec{h_{m_i}}$ denotes the feature of the *i*-th molecule extracted from its conformer $C_i$, and SchNet is a variant of Deep Tensor Neural Network (DTNN). The SchNet model incorporates a continuous-filter convolution layer, which is particularly well-suited for molecular dynamics simulations aimed at predicting potential energy surfaces and energy-conserving force fields. A SchNet model can be formulated as follows:

10 $$\begin{aligned} \begin{aligned} \varvec{e_{u}^{0}}&= \text {embedding}\left( \varvec{I_{u}}\right) , \\ \varvec{e_{u}^{\ell }}&= \text {MLP}\left( \sum _{v}f\left( \varvec{e_{v}^{\ell -1}}, r_{u}, r_{v}\right) \right) , \\ \varvec{e_{u}^K}&= \text {MLP}\left( \varvec{e_{u}^{K-1}}\right) , \\ \varvec{h_{m_i}}&= \frac{1}{n}\sum _u \varvec{e_u^K}, \end{aligned} \end{aligned}$$

where $\varvec{I_u}$ denotes the input feature of atom *u* for the embedding layer, $\varvec{e_u^\ell }$ denotes the output at layer $\ell$ ($0<\ell <K$) for atom *u*, *r* denotes the spatial coordinate of the corresponding atom, MLP denotes the multi-layer perceptron, *K* denotes the number of hidden layers, and

11 $$\begin{aligned} \begin{aligned} f\left( \varvec{e_{v}}, r_{u}, r_{v}\right) = \varvec{e_{v}} \cdot \exp \left( -\gamma \left\| \Vert r_{v}-r_{u}\Vert _{2}-\mu _k\right\| _{2}^{2}\right) \end{aligned} \end{aligned}$$

is the continuous-filter convolution layer that captures continuous coordinates of atoms instead of discrete ones using different hyper-parameters $\gamma$ and $\mu _k$.

## Results and discussion

### Experimental settings

ReaMVP is implemented in Python (version 3.10). It uses RDKit (version 2022.9.5) [40] for reaction preprocessing and SMILES validation, and employs Pytorch (version 2.0.0) [55] and DGL (version 1.0.2) [56] for sequence and conformer modeling.

We use three metrics, namely mean absolute error (MAE), root mean squared error (RMSE), and coefficient of determination (R^2^), to assess the performance of yield prediction. The sequence encoder comprises an embedding layer with a dimensionality of 256, followed by a two-layer bidirectional GRU model with a hidden layer of 128-D and a dropout ratio of 0.3. The conformer encoder employs a SchNet model (detailed initial features of atoms are provided in Additional file 1: Table S7) with four interaction blocks, 64 Gaussian filters, a molecular radius threshold of ten, and all hidden layers and filters with a dimension of 128. Similarly, a two-layer bidirectional GRU model is employed with a hidden layer of 128-D and a dropout ratio of 0.3. We use the Adam [57] optimizer with default parameters during the training process. In the pre-training stage I, we set $\lambda$ to 1.0 after exploring values in {0.1, 0.5, 1.0} (see “Both Align and Contrast operations are indispensable for the self-supervised pre-training” Section for details). In the pre-training stage II, we use a regression head that takes the concatenation of the outputs from the sequence encoder and conformer encoder (see “Multi-view learning excels over single-view methods on most splits” Section for details). During the fine-tuning stage, we perform a grid search for hyper-parameters, including the learning rate in {3e–4, 1e–3, 3e–3}, the dropout ratio in {0.1, 0.3}, the weight decay in {0, 1e–4, 1e–5}, and the loss function in {MSE, MAE}.

**Table 3:** Results of the Buchwald-Hartwig dataset under ligand-based **out-of-sample conditions**

Split type	Measure	YieldBERT	YieldBERT-DA	UA-GNN	ReaMVP
Test 1	MAE	$7.351\pm 0.099$	$\mathbf {7.015\pm 0.758}$	$8.082\pm 0.827$	$7.276\pm 0.124$
	RMSE	$11.441\pm 0.342$	$11.761\pm 1.398$	$13.746\pm 1.175$	$\mathbf {10.768\pm 0.136}$
	R^2^	$0.824\pm 0.010$	$0.811\pm 0.047$	$0.744\pm 0.042$	$\mathbf {0.844\pm 0.004}$
Test 2	MAE	$7.266\pm 0.724$	$6.588\pm 0.328$	$6.300\pm 0.647$	$\mathbf {6.078\pm 0.149}$
	RMSE	$11.144\pm 1.267$	$9.886\pm 0.741$	$9.476\pm 1.027$	$\mathbf {8.722\pm 0.179}$
	R^2^	$0.829\pm 0.037$	$0.866\pm 0.020$	$0.876\pm 0.026$	$\mathbf {0.896\pm 0.004}$
Test 3	MAE	$9.129\pm 0.745$	$11.052\pm 0.950$	$8.986\pm 0.314$	$\mathbf {8.969\pm 0.491}$
	RMSE	$14.276\pm 0.820$	$18.041\pm 1.395$	$14.939\pm 0.622$	$\mathbf {12.791\pm 0.769}$
	R^2^	$0.741\pm 0.030$	$0.585\pm 0.067$	$0.717\pm 0.024$	$\mathbf {0.792\pm 0.025}$
Test 4	MAE	$13.671\pm 1.067$	$18.422\pm 0.620$	$13.190\pm 0.754$	$\mathbf {10.605\pm 0.656}$
	RMSE	$19.679\pm 1.397$	$24.279\pm 0.494$	$18.774\pm 0.566$	$\mathbf {14.618\pm 0.932}$
	R^2^	$0.444\pm 0.077$	$0.157\pm 0.034$	$0.496\pm 0.031$	$\mathbf {0.693\pm 0.038}$
Plate 1	MAE	$10.036\pm 0.300$	$\mathbf {8.880\pm 0.552}$	$10.981\pm 0.624$	$9.576\pm 0.299$
	RMSE	$14.832\pm 0.367$	$\mathbf {13.697\pm 0.432}$	$15.467\pm 1.045$	$13.808\pm 0.372$
	R^2^	$0.752\pm 0.012$	$\mathbf {0.789\pm 0.013}$	$0.730\pm 0.037$	$0.785\pm 0.011$
Plate 2	MAE	$16.822\pm 1.988$	$\mathbf {14.449\pm 0.375}$	$15.547\pm 1.004$	$14.651\pm 1.777$
	RMSE	$21.711\pm 2.283$	$19.682\pm 0.342$	$21.479\pm 1.617$	$\mathbf {19.356\pm 2.003}$
	R^2^	$0.181\pm 0.171$	$0.334\pm 0.023$	$0.202\pm 0.121$	$\mathbf {0.349\pm 0.129}$
Plate 3	MAE	$9.932\pm 0.287$	$10.796\pm 1.016$	$\mathbf {8.163\pm 0.570}$	$8.855\pm 0.537$
	RMSE	$13.714\pm 0.341$	$14.788\pm 1.287$	$\mathbf {11.901\pm 0.635}$	$12.139\pm 0.479$
	R^2^	$0.718\pm 0.014$	$0.669\pm 0.056$	$\mathbf {0.787\pm 0.023}$	$0.779\pm 0.017$
Plate 2 new	MAE	$12.629\pm 1.259$	$11.521\pm 0.495$	$12.546\pm 1.071$	$\mathbf {10.322\pm 0.556}$
	RMSE	$17.509\pm 1.917$	$16.540\pm 0.271$	$18.568\pm 1.387$	$\mathbf {13.987\pm 0.583}$
	R^2^	$0.508\pm 0.106$	$0.566\pm 0.014$	$0.451\pm 0.083$	$\mathbf {0.689\pm 0.026}$

Bold entries highlight the best performance

### ReaMVP demonstrates superior generalization capability compared to SOTA models

To evaluate the effectiveness of ReaMVP, we compare its performance with three state-of-the-art DL yield prediction methods as listed below.

- YieldBERT [3] adapts a pre-trained BERT encoder [19] to predict chemical reaction yields via reaction SMILES.
- YieldBERT-DA [24] is an extension of YieldBERT, which uses the same pre-trained BERT encoder and adds SMILES randomization and permutation as data augmentation.
- UA-GNN [25] aggregates molecular embeddings learned by graph neural networks using a set of molecular graphs with permutation invariance and utilizes uncertainty-aware learning and inference.

**Table 4:** Results of the Suzuki-Miyaura dataset under ligand-based **out-of-sample conditions**

Split type	Measure	YieldBERT	YieldBERT-DA	UA-GNN	ReaMVP
Test 1	MAE	$19.357\pm 0.174$	$19.813\pm 0.177$	$16.328\pm 0.588$	$\mathbf {15.186\pm 0.492}$
	RMSE	$25.000\pm 0.095$	$24.975\pm 0.210$	$21.996\pm 0.818$	$\mathbf {19.564\pm 0.742}$
	R^2^	$0.306\pm 0.005$	$0.307\pm 0.012$	$0.462\pm 0.400$	$\mathbf {0.574\pm 0.033}$
Test 2	MAE	$14.845\pm 0.364$	$15.777\pm 0.239$	$15.587\pm 0.356$	$\mathbf {13.905\pm 0.286}$
	RMSE	$19.592\pm 0.386$	$19.639\pm 0.264$	$20.485\pm 0.391$	$\mathbf {18.357\pm 0.349}$
	R^2^	$0.469\pm 0.021$	$0.467\pm 0.014$	$0.420\pm 0.022$	$\mathbf {0.534\pm 0.018}$
Test 3	MAE	$15.438\pm 0.286$	$15.235\pm 0.492$	$13.624\pm 0.119$	$\mathbf {13.518\pm 0.284}$
	RMSE	$20.051\pm 0.371$	$19.455\pm 0.389$	$19.090\pm 0.342$	$\mathbf {18.236\pm 0.294}$
	R^2^	$0.357\pm 0.024$	$0.395\pm 0.025$	$0.417\pm 0.021$	$\mathbf {0.468\pm 0.017}$
Test 4	MAE	$18.862\pm 0.095$	$18.644\pm 0.082$	$\mathbf {15.613\pm 0.382}$	$15.985\pm 0.615$
	RMSE	$23.114\pm 0.119$	$23.726\pm 0.141$	$22.176\pm 0.270$	$\mathbf {21.796\pm 0.700}$
	R^2^	$0.239\pm 0.008$	$0.229\pm 0.010$	$0.299\pm 0.017$	$\mathbf {0.323\pm 0.043}$

Bold entries highlight the best performance

To perform a rigorous assessment of model generalization capability to unseen data, we experiment with the out-of-sample splits (as described in “Downstream datasets” Section), i.e., the reactions for test include molecules that are not present in the training set. The Buchwald-Hartwig (BH) dataset has eight ligand-based splits (Tests 1–4, Plates 1–3, and Plate 2 new) and five reactant-based splits (Halide Br, Halide Cl, Halide I, Pyridyl, and Nonpyridyl), while the Suzuki-Miyaura (SM) dataset has four ligand-based splits (Tests 1–4).

Tables 3, 4 present the average results from ligand-based splits across five random runs for the BH and SM datasets, respectively. The results of Tests 1–4 for the BH dataset are as reported in the original papers of the three baseline models; while the results of other splits are not available, thus we reproduce the three models using their released codes and conduct the experiments. To reproduce YieldBERT and YieldBERT-DA, we employ the pre-trained model labeled as “pre-trained” instead of “ft”. During the fine-tuning, we determine the hyper-parameters via a grid-search, including the learning rate in {5e-6, 1e-5, 5e-5, 1e-4} and the dropout ratio in {0.3, 0.4, 0.5, 0.6, 0.7}. For the augmentation hyper-parameters of YieldBERT-DA, we adopt the fixed random order with a random type of “rotated” and conduct ten permutations for training and testing the model. To reproduce UA-GNN, we maintain the same set of hyper-parameters as specified in the original paper to ensure consistency and comparability.

**Table 5:** Results of the Buchwald-Hartwig dataset under reactant-based **out-of-sample conditions**

Split type	Measure	YieldBERT	YieldBERT-DA	UA-GNN	ReaMVP
Halide Br	MAE	$7.882\pm 0.311$	$8.431\pm 0.415$	$7.336\pm 0.824$	$\mathbf {7.118\pm 0.873}$
	RMSE	$11.180\pm 0.379$	$12.457\pm 0.508$	$10.185\pm 1.334$	$\mathbf {10.034\pm 1.126}$
	R^2^	$0.803\pm 0.013$	$0.756\pm 0.020$	$0.834\pm 0.044$	$\mathbf {0.840\pm 0.037}$
Halide Cl	MAE	$18.727\pm 2.130$	$17.769\pm 0.735$	$26.822\pm 1.243$	$21.664\pm 1.588$
	RMSE	$25.184\pm 2.781$	$21.253\pm 0.571$	$33.169\pm 0.783$	$25.881\pm 0.936$
	R^2^	$-0.434\pm 0.316$	$-0.010\pm 0.054$	$-1.459\pm 0.117$	$-0.498\pm 0.107$
Halide I	MAE	$10.359\pm 0.422$	$9.201\pm 0.419$	$15.950\pm 2.924$	$\mathbf {8.877\pm 0.254}$
	RMSE	$14.388\pm 0.398$	$\mathbf {12.419\pm 0.631}$	$20.793\pm 3.359$	$13.084\pm 0.314$
	R^2^	$0.676\pm 0.018$	$\mathbf {0.758\pm 0.025}$	$0.307\pm 0.225$	$0.732\pm 0.013$
Pyridyl	MAE	$17.443\pm 1.009$	$18.406\pm 0.480$	$\mathbf {16.946\pm 0.214}$	$17.172\pm 0.833$
	RMSE	$23.904\pm 1.126$	$26.300\pm 0.328$	$24.819\pm 0.632$	$\mathbf {21.401\pm 1.147}$
	R^2^	$0.350\pm 0.060$	$0.215\pm 0.020$	$0.301\pm 0.035$	$\mathbf {0.479\pm 0.056}$
Nonpyridyl	MAE	$\mathbf {14.143\pm 0.684}$	$15.043\pm 0.351$	$18.802\pm 1.216$	$17.259\pm 1.320$
	RMSE	$19.075\pm 0.751$	$\mathbf {18.580\pm 0.244}$	$23.610\pm 2.059$	$21.171\pm 1.141$
	R^2^	$0.308\pm 0.055$	$\mathbf {0.344\pm 0.017}$	$-0.067\pm 0.188$	$0.146\pm 0.090$

Bold entries highlight the best performance

Remarkably, we observe that ReaMVP demonstrates superior performance under various evaluation metrics in most cases, except for the BH Plate 1 split and the BH Plate 3 split. The proposed model exhibits outstanding performance in out-of-sample yield prediction tasks. For instance, the R^2^ value of ReaMVP increases by approximately 40% under the BH Test 4 split, by approximately 22% under the BH Plate 2 new split, and by approximately 24% under the SM Test 1 split. These substantial improvements in prediction performance highlight the effectiveness of distribution alignment during the pre-training stage, which enhances the generalization capability of models. ReaMVP demonstrates a more balanced representation of isoxazole additives in the BH dataset and ligands in the SM dataset, as evidenced by its consistent performance across these different categories.

In addition to the ligand-based splits, we experiment with five reactant-based splits of the BH dataset. Table 5 presents the average results along with the corresponding standard deviations of five random runs. ReaMVP yields the best performance on the Halide Br and Pyridyl splits, while also attaining a top 2 position (on par with YieldBERT-DA) for the Halide I split.

We also apply the random forest (RF) models and support vector machines (SVMs) on the BH dataset under out-of-sample conditions to offer a better understanding of comparisons between different methods (detailed values are shown in Additional file 1: Table S5). Reaction features are adopted from Mandana Saebi et al. [12]. We observe that non-DL methods perform worse in most cases and have a higher risk of overfitting, especially for reactant-based splits.

**Table 6:** Results of the Buchwald-Hartwig and Suzuki-Miyaura datasets with random splits

Dataset	Measure	YieldBERT	YieldBERT-DA	UA-GNN	ReaMVP
Buchwald-Hartwig	MAE	$3.990\pm 0.153$	$3.090\pm 0.118$	$\mathbf {2.920\pm 0.056}$	$3.108\pm 0.071$
	RMSE	$6.014\pm 0.272$	$4.799\pm 0.261$	$\mathbf {4.433\pm 0.085}$	$4.626\pm 0.139$
	R^2^	$0.951\pm 0.005$	$0.969\pm 0.004$	$\mathbf {0.974\pm 0.001}$	$0.971\pm 0.002$
Suzuki-Miyaura	MAE	$8.128\pm 0.344$	$6.598\pm 0.270$	$\mathbf {6.116\pm 0.223}$	$6.587\pm 0.195$
	RMSE	$12.073\pm 0.463$	$10.524\pm 0.482$	$\mathbf {9.467\pm 0.459}$	$10.367\pm 0.423$
	R^2^	$0.815\pm 0.013$	$0.859\pm 0.012$	$\mathbf {0.886\pm 0.010}$	$0.864\pm 0.010$

Bold entries highlight the best performance

Notably, both the DL and non-DL methods underperform on the Halide Cl split with negative values of R^2^. In contrast, most models obtain meaningful predictions for the Halide Br and Halide I splits. To further investigate the inferior performance for the Halide Cl split among three halide-based splits, we analyze the yield distributions of the training dataset alongside the corresponding test dataset (detailed distributions are present in Additional file 1: Figure S2). Three histogram metrics are computed to quantify the dissimilarity between these distributions (detailed values are present in Additional file 1: Table S1). The results reveal a substantial dissimilarity in yield distributions between the training and test datasets for the Halide Cl split. For instance, the normalized histogram intersection decreases by roughly 33%, the chi-squared distance increases by around 91%, and the Jeffreys divergence increases by approximately 216% in comparison with the Halide I split. Such a distribution shift poses a great challenge for machine learning models to accurately predict reaction yields.

Additionally, as many previous studies reported results under random conditions, here we also compare the results obtained by random splitting. Table 6 presents the averaged results with standard deviations for ten random 70/30 splits. To ensure a fair comparison, all methods utilize the same random splits. ReaMVP exhibits competitive performance compared to the state-of-the-art method by a slight margin. YieldBERT, YieldBERT-DA, and ReaMVP all utilize large-scale pre-training strategies followed by fine-tuning on downstream tasks. These methods are supposed to have a higher generalization capability than those without pre-training. ReaMVP generally achieves the best performance among them, except for MAE in the BH dataset. It suggests that considering more dimensional information during pre-training is an effective approach to improving model performance.

Under random splits, UA-GNN, the model without pre-training, outperforms the three models with pre-training, which may be attributed to the presence of data leakage in the datasets. Both the BH and SM datasets are characterized by a relatively small number of unique molecules, consisting of only 51 and 36 molecules, respectively. Hence the training set is likely to contain all molecules at least once under the random 70/30 split setting [15]. Overlapping data between the training and test sets poses significant challenges in accurately evaluating a model’s generalization ability. As can be seen from Table 6, the models exhibit notably better performance under random conditions compared to out-of-sample conditions. Specifically, the BH dataset shows exceptionally high R^2^ values exceeding 0.95, which is remarkably high in practical scenarios. This suggests that the model performance may be overestimated when using random splits, potentially leading to misleading conclusions.

**Fig. 4:**
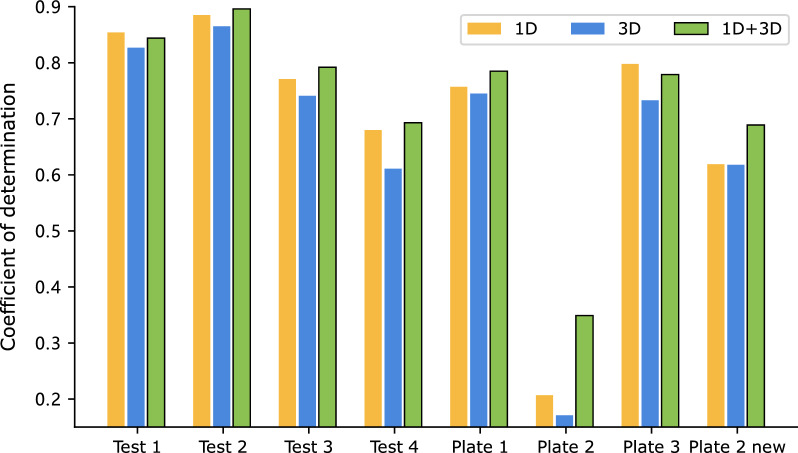
R^2^ values of the Buchwald-Hartwig dataset under eight out-of-sample conditions based on ligands with different data views for fine-tuning

### Multi-view learning excels over single-view methods on most splits

ReaMVP utilizes both the 1D sequential and 3D geometric information of molecules to learn the representation of chemical reactions. To investigate the impact of the data view for the predictor, we set $\lambda$ (in Eq. 6) to 1.0 and examine the performance when considering the sequence encoder alone, the conformer encoder alone, and the concatenation of their outputs both during the pre-training stage II and the fine-tuning stage.

**Fig. 5:**
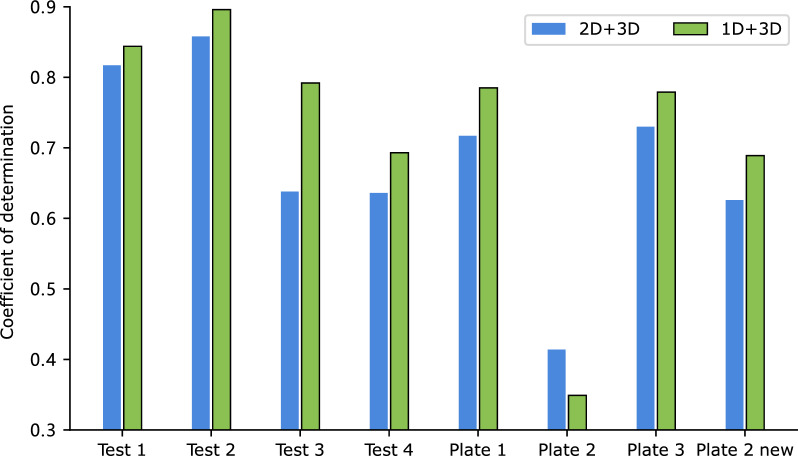
R^2^ values of the Buchwald-Hartwig dataset under eight out-of-sample conditions based on ligands with different data views for pre-training and fine-tuning

Figure 4 (detailed values are shown in Additional file 1: Table S2) depicts the results. Taking the concatenation of outputs from each encoder yields the best performance against others in six out of eight splits, with the remaining two splits showing very close results to the best ones. Notably, on Plate 2 and Plate 2 new, the ‘1D$+$3D’ approach substantially improves performance, with an increase of over 0.14 and 0.07 in R^2^ against 1D- and 3D-only methods, respectively.

The results highlight the efficacy of integrating information from multiple views to enhance model performance. Interestingly, the method using 3D information exhibits inferior performance compared to its 1D counterpart, possibly because some 3D coordinates of molecules generated by the ETKDG algorithm in RDKit are inaccurate. Obtaining precise 3D geometric information is a challenging task. Nonetheless, the simulated 3D information can still provide valuable supplementary information to the 1D sequence and ultimately improve the accuracy.

Additionally, we further investigate the influence of multi-view choices. The ReaMVP model includes a sequence encoder and a conformer encoder to encode chemical reactions during the pre-training phase. The model allows for various data views, including 1D sequential, 2D topologic, and 3D geometric data structures. We replace the sequence encoder with a graph encoder (the graph neural network model GIN [58]) to include 2D features from consideration. This adjustment transforms the input into molecular graph structures, where atoms and bonds are treated as nodes and edges, respectively.

**Fig. 6:**
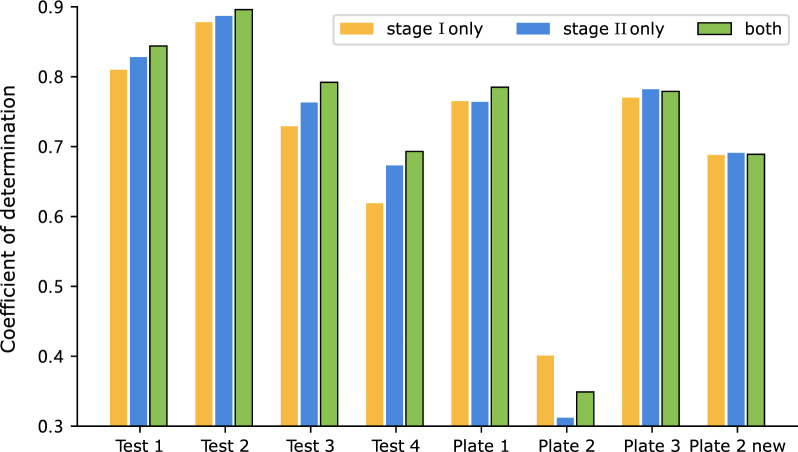
R^2^ values of the Buchwald-Hartwig dataset under eight out-of-sample conditions based on ligands with different pre-training strategies

Figure 5 (detailed values are given in Additional file 1: Table S2) presents the results. The ‘1D$+$3D’ approach yields the better performance in seven out of eight splits, exhibiting an average increase of about 0.05 in R^2^. The superior results of ‘1D$+$3D’ over ‘2D$+$3D’ can be attributed from complementarity between views and the nature of contrastive learning. First, considering the differences in these representations, the 1D SMILES and 3D conformer views could provide more complementary information to each other than the 2D and 3D views. The 1D SMILES view encapsulates bonding and atomic information effectively in a compact form, while the 3D conformer view provides spatial information. By contrast, there is an overlap between the information provided by the 3D and 2D structures, which may lead to shortcut learning and reduce the model’s generalization capability. Second, in contrastive learning, an essential requirement is data variance. It is possible that the combination of 1D and 3D creates more significant variance, leading the contrastive learning models to work more reliably, thereby enhancing their overall performance.

### Both self-supervised and supervised pre-training enhance prediction performance

ReaMVP includes two stages of pre-training. We refer to stage I as self-supervised pre-training since the model is trained without yield information, and stage II as supervised pre-training, as the training data of stage II does not overlap with the downstream datasets (i.e., no shared reactions), yet the pre-training task also involves yield prediction.

**Fig. 7:**
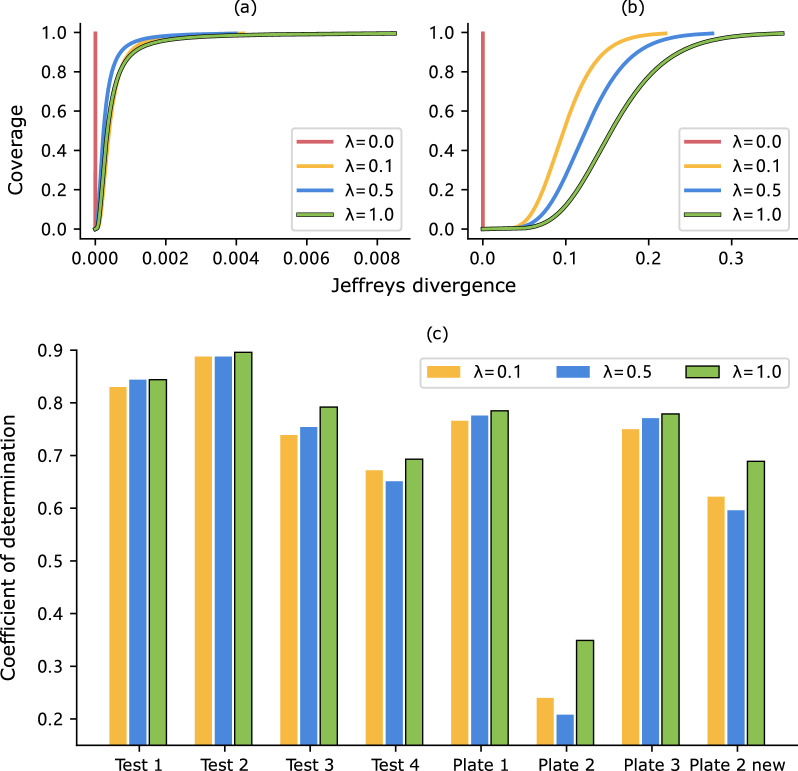
Experimental results on investigating the efficacy of the pre-training stage I. **a** The CDF curves of positive pairs. **b** The CDF curves of negative pairs. **c** R^2^ values of the Buchwald-Hartwig dataset under eight out-of-sample conditions based on ligands with different weighting coefficients

Here, we conduct experiments that only employ the first or second stage of pre-training. The results shown in Fig. 6 demonstrate the efficacy of pre-training stages I and II. Specifically, it leads to inferior performance in seven out of eight splits with only stage I and in six out of eight splits with only stage II. Note that since there is no overlap between the reactions in the two-stage pre-training data and the downstream data, we can conclude that both the self-supervised and supervised pre-training, which primarily include reaction types different from those in the downstream data, capture some common patterns related to reaction mechanisms. As a result, the two-stage pre-training helps improve yield prediction for the downstream datasets.

### Both Align and Contrast operations are indispensable for the self-supervised pre-training

During our pre-training stage I, there are two basic operations, i.e., Align and Contrast (as shown in Fig. 2). The process of distribution alignment (Align), achieved through the utilization of Kullback–Leibler divergence, primarily focuses on positive pairs that correspond to different views of the same reactions. While pulling the distributions between positive pairs closer together is a crucial aspect, it is equally important to differentiate the outputs between distinct views from different sample pairs to avoid trivial representations. To investigate the impact of distribution alignment and contrastive learning using the concatenation of outputs from the sequence encoder and the conformer encoder, we experiment with different weighting coefficients in Equation (6).

Figure 7c (detailed values are given in Additional file 1: Table S3) presents the R^2^ values obtained on downstream datasets under out-of-sample conditions when $\lambda$ equals 0.1, 0.5, and 1.0, respectively. We set the batch size as 32 and extract reaction pairs from the pre-training dataset USPTO-2 to compute the Jeffreys divergence of positive and negative pairs. Figure 7a and  b reveal the cumulative distribution function (CDF) curves for both the positive pairs and the negative pairs.

Based on the results, there are some observations that can be made:

1. The use of distribution alignment via Kullback–Leibler divergence alone may not produce the desired outcome. When the hyper-parameter $\lambda$ is set to 0, both the distances between positive and negative pairs in the alignment space tend to be zero, which can limit the model’s ability to capture meaningful patterns in the data.
2. Using distribution alignment along with contrastive learning proves to be an effective approach to learning more informative representations that yield better performance on downstream tasks, especially under out-of-sample conditions.
3. Different weighting coefficients have a slight influence on the Jeffreys divergence between positive pairs but exhibit a more significant impact on negative pairs.
4. The trends observed in the CDF curves are generally consistent with the downstream performance, which indicates that the Jeffreys divergence may serve as a criterion for model selection during pre-training. We notice that it outperforms others in all cases when $\lambda$ equals 1.0. This setting effectively enforces a notable separation between negative pairs and maintains the proximity between positive pairs. Meanwhile, results obtained with $\lambda$ equal to 0.5 tend to surpass those obtained with $\lambda$ equal to 0.1.

### Further investigation on predicting the data from electronic laboratory notebooks (ELNs)

The BH dataset and SM dataset are both from high-throughput experiments (HTE), yet they represent a small part of the reaction space due to limited categories of molecules. For example, the BH dataset and the SM dataset form only five and one different products, respectively. Such a low diversity may lead to obstacles for a general-purpose reaction yield prediction [59].

To evaluate the model’s performance on previously unseen and complex data points, we conduct experiments on the ELN BH dataset, which was released by Mandana Saebi et al. [12]. They collected a legacy dataset for Buchwald-Hartwig reactions from electronic laboratory notebooks with a wide range of substrates, ligands, and solvents. The structural diversity of the ELN dataset is much higher than that of the HTE dataset. We apply the same random 70/30 split as reported in the original paper.

The results are present in Additional file 1: Table S6. We observe that ReaMVP outperforms other DL methods in all metrics, demonstrating a higher generalization capability. However, the non-DL model, RF with RDKit features, yields the best performance. Besides, none of the models provide meaningful predictions. Our findings are in agreement with recent studies on reaction condition prediction [12, 60]. While DL models excel on larger datasets BH and SM, their relatively inferior performance on the smaller ELN dataset warrants examination. One potential explanation is that DL models tend to perform well when trained on larger, more comprehensive datasets, which allow them to learn representations sufficiently. On smaller datasets like ELN BH, they might be overfitting to noise or unable to develop a rich representation due to the scarcity of data, resulting in suboptimal performance. Moreover, the structural diversity introduced by a wide range of substrates, ligands, bases, and solvents in the ELN BH dataset poses a significant challenge to machine learning models. Although the pre-training techniques are employed, due to the large gap between the pre-training data and the downstream task data, the small amount of fine-tuning data leads to poor generalization capability.

## Conclusions

In this study, we introduce ReaMVP, a large-scale multi-view pre-training method with two pre-training stages designed to enhance the representation of chemical reactions for predicting reaction yields. In the first pre-training stage, we learn representations of reactions by learning the consistency relationship between different views of reactions via distribution alignment and contrastive learning. In the second pre-training stage, we combine the outputs from the sequence encoder and conformer encoder and incorporate a predictor for supervised pre-training, thereby further refining the learned representations for accurate yield prediction.

While the ETKDG algorithm used to obtain molecular coordinates may generate inaccurate 3D information, the experimental findings demonstrate the effectiveness of the incorporation of the 3D view. By combining 1D and 3D representations, we can capitalize on the strengths of both views and mitigate their limitations, leading to enhanced performance in predicting chemical reaction yields. Notably, even when the algorithm fails to simulate 3D structures, ReaMVP’s sequence encoder can still effectively predict chemical reaction yields.

ReaMVP stands out from the state-of-the-art methods with its exceptional performance in out-of-sample scenarios, demonstrating its potential in predicting chemical reaction yields involving unseen additives or ligands. Additionally, the model can be extended to other prediction tasks related to chemical reaction outcomes. With its robust performance and versatility, ReaMVP represents a valuable tool for chemists and researchers in chemical reaction studies.

### Supplementary information

**Additional file 1: Figure S1.** Out-of-sample splits of the Buchwald-Hartwig dataset based on reactants. **Figure S2.** Yield distributions of the halide-based splits of the Buchwald-Hartwig dataset. **Figure S3.** Out-of-sample splits of the Suzuki-Miyaura dataset based on ligands.**Figure S4.** Yield distribution of the pre-training dataset. **Table S1.** Histogram metrics between the training and test yield distributions of the Buchwald-Hartwig dataset based on aryl halides. **Table S2–S6.** Detailed machine learning predictions. **Table S7.** Initial features of atoms in the SchNet model.

## Data Availability

The pre-training dataset we used is collected from USPTO (https://figshare.com/articles/dataset/Chemical_reactions_from_US_patents_1976-Sep2016_/5104873) and CJHIF (https://github.com/jshmjs45/data_for_chem). The Buchwald-Hartwig and Suzuki-Miyaura datasets are publicly accessible from https://github.com/rxn4chemistry/rxn_yields/. Our code and the out-of-sample splits Tests 1–4 of the Suzuki-Miyaura dataset are available at https://github.com/Meteor-han/ReaMVP.

## Abbreviations

DL: Deep learning
SMILES: Simplified molecular-input line-entry system
SMARTS: SMILES arbitrary target specification
USPTO: United States Patent and Trademark Office
CJHIF: Chemical journals with high impact factor
OPSIN: Open parser for systematic IUPAC nomenclature
CIR: Chemical identifier resolver
RNN: Recurrent neural network
NLP: Natural language processing
DTNN: Deep tensor neural network
GRU: Gated recurrent unit
MAE: Mean absolute error
RMSE: Root-mean-square error
R^2^: Coefficient of determination
ELNs: Electronic laboratory notebooks
BH: Buchwald-Hartwig
SM: Suzuki-Miyaura
CDF: Cumulative distribution function
