# Supplementary material for: Prediction of chemical reaction yields with large-scale multi-view pre-training
Source: J Cheminform. 2024 Feb 25;16:22. doi: 10.1186/s13321-024-00815-2 (PMC10895839; doi:10.1186/s13321-024-00815-2)
Supplement: Supplementary file 1 — Additional file 1: Figure S1. Out-of-sample splits of the Buchwald-Hartwig dataset based on reactants. Figure S2. Yield distributions of the halide-based splits of the Buchwald-Hartwig dataset. Figure S3. Out-of-sample splits of the Suzuki-Miyaura dataset based on ligands.Figure S4. Yield distribution of the pre-training dataset. Table S1. Histogram metrics between the training and test yield distributions of the Buchwald-Hartwig dataset based on aryl halides. Table S2–S6. Detailed machine learning predictions. Table S7. Initial features of atoms in the SchNet model. [file 13321_2024_815_MOESM1_ESM.pdf]

Supporting Information for Prediction of Chemical Reaction Yields with Large-Scale Multi-View Pre-training

Runhan Shi,† Gufeng Yu,† Xiaohong Huo,‡ and Yang Yang*,†

†*Department of Computer Science and Engineering, and Key Laboratory of Shanghai Education Commission for Intelligent Interaction and Cognitive Engineering, Shanghai Jiao Tong University, Shanghai 200240, China*

‡*Shanghai Key Laboratory for Molecular Engineering of Chiral Drugs, Frontiers Science Center for Transformative Molecules, School of Chemistry and Chemical Engineering, Shanghai Jiao Tong University, Shanghai 200240, China*

E-mail: yangyang@cs.sjtu.edu.cn

Contents

1 Out-of-sample splits of the Buchwald-Hartwig dataset based on reactants 2

2 Yield distributions of the halide-based splits of the Buchwald-Hartwig dataset 3

3 Out-of-sample splits of the Suzuki-Miyaura dataset based on ligands 4

4 Results of machine learning predictions 5

4.1 Results of ablation study for the multi-view learning 5

4.2 Results of ablation study for the pre-training stages 6

4.3 Results of ablation study on Align and Contrast operations for the self-supervised pre-training 6
4.4 Results of non-deep-learning methods 7
4.5 Results of the data from electronic laboratory notebooks 7

5 Initial features of atoms in the SchNet model 8

6 Yield distribution of the pre-training dataset 8

1 Out-of-sample splits of the Buchwald-Hartwig dataset based on reactants

There are a total of 15 aryl halides, almost equally distributed among the Buchwald-Hartwig dataset [8]. We extract the same halide types or pyridyl types as a group with their corresponding reactions to construct the test set and take the remaining reactions as the training set. Hence, we obtain five splits in total, labeled as Halide Br, Halide Cl, Halide I, Pyridyl, and Nonpyridyl. Figure S1 shows the structure of each aryl halide and the splits.

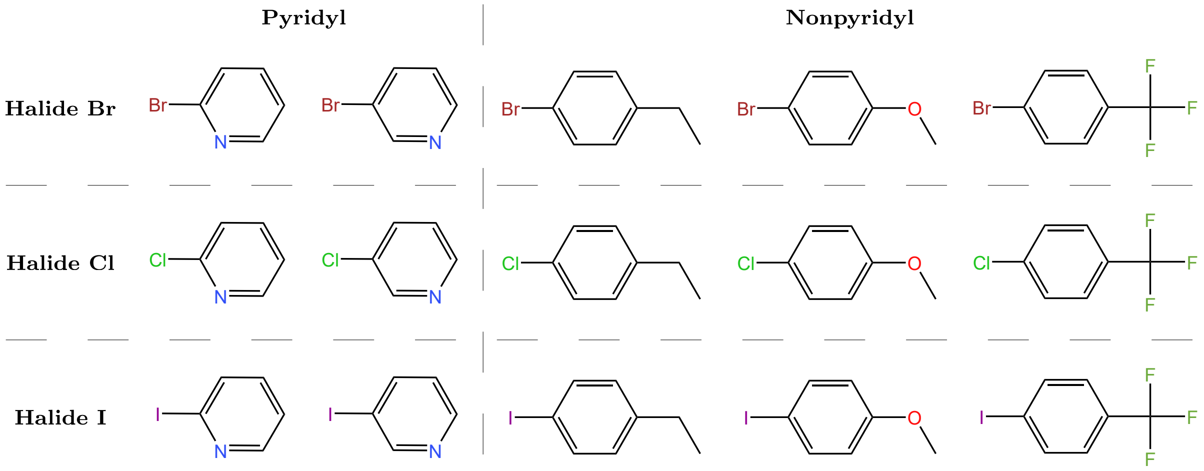

Figure S1: Reactants (aryl halides) and the corresponding groups under out-of-sample conditions (Halide Br, Halide Cl, Halide I, Pyridyl, and Nonpyridyl) of the Buchwald-Hartwig dataset.

2 Yield distributions of the halide-based splits of the Buchwald-Hartwig dataset

For the Halide Br, Cl, and I splits, we set the number of bins to 100 and plot the yield distribution of the training dataset and test dataset shown in Figure S2. To evaluate the distribution similarity, the normalized histogram intersection (Intersection, the lower, the less similar), chi-squared distance (Chi-squared, the higher, the less similar), and Jeffreys divergence (Divergence, the higher, the less similar) are calculated between the training yield distribution and the corresponding test yield distribution after converting counts to probabilities, as shown in Equation (1).

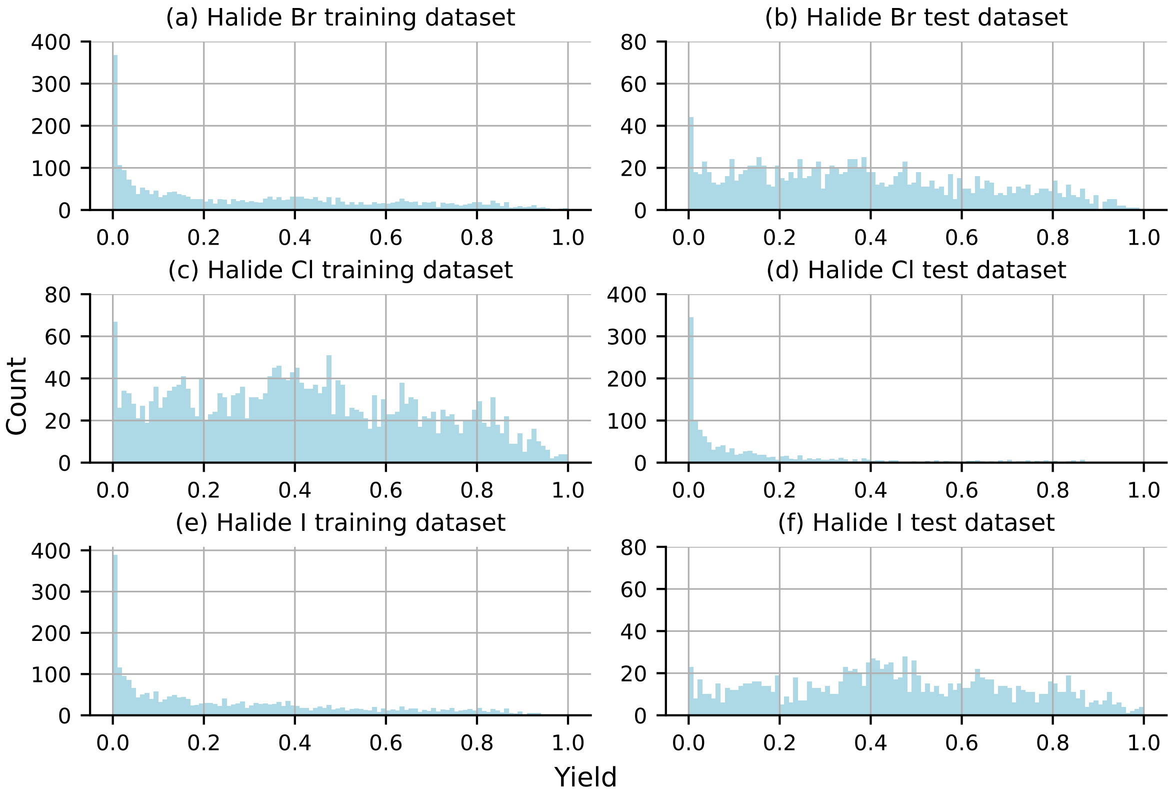

Figure S2: Yield distributions of halide-based splits.

$$\text{Intersection} = \frac{\sum_{i=1}^{n_{\text{bins}}} \min\left(p_i^{\text{training}}, p_i^{\text{test}}\right)}{\sum_{i=1}^{n_{\text{bins}}} \max\left(p_i^{\text{training}}, p_i^{\text{test}}\right)},$$

$$\text{Chi-Squared} = \sum_{i=1}^{n_{\text{bins}}} \frac{(p_i^{\text{training}} - p_i^{\text{test}})^2}{p_i^{\text{training}} + p_i^{\text{test}}}, \tag{1}$$

$$\text{Divergence} = \frac{1}{2}\sum_{i=1}^{n_{\text{bins}}} (p_i^{\text{training}} \log \frac{p_i^{\text{training}}}{p_i^{\text{test}}} + p_i^{\text{test}} \log \frac{p_i^{\text{test}}}{p_i^{\text{training}}}).$$

Table S1 presents the results.

Table S1: Histogram metrics between the training and test yield distributions of the Buchwald-Hartwig dataset based on aryl halides. Bold entries denote the highest dissimilarity.

Split type	Intersection	Chi-Squared	Divergence ($1e-6$)
Halide Br	0.632	0.166	0.756
Halide Cl	**0.315**	**0.674**	**3.918**
Halide I	0.467	0.352	1.238

3 Out-of-sample splits of the Suzuki-Miyaura dataset based on ligands

There are a total of 12 ligands (if consider the "None" ligand), equally distributed among the Suzuki-Miyaura dataset [46]. Each ligand is involved in 480 reactions. Inspired by the out-of-sample splits of the Buchwald-Hartwig dataset [8], we extract three ligands as a group with their corresponding reactions to construct the test set and take the remaining reactions as the training set. Hence we obtain four splits in total, labeled as Tests 1-4. Each test set consists of 1440 reactions. Figure S3 shows the structure of each ligand and the splits of Tests 1-4. Note that we put structurally similar ligands together to make the problem more challenging.

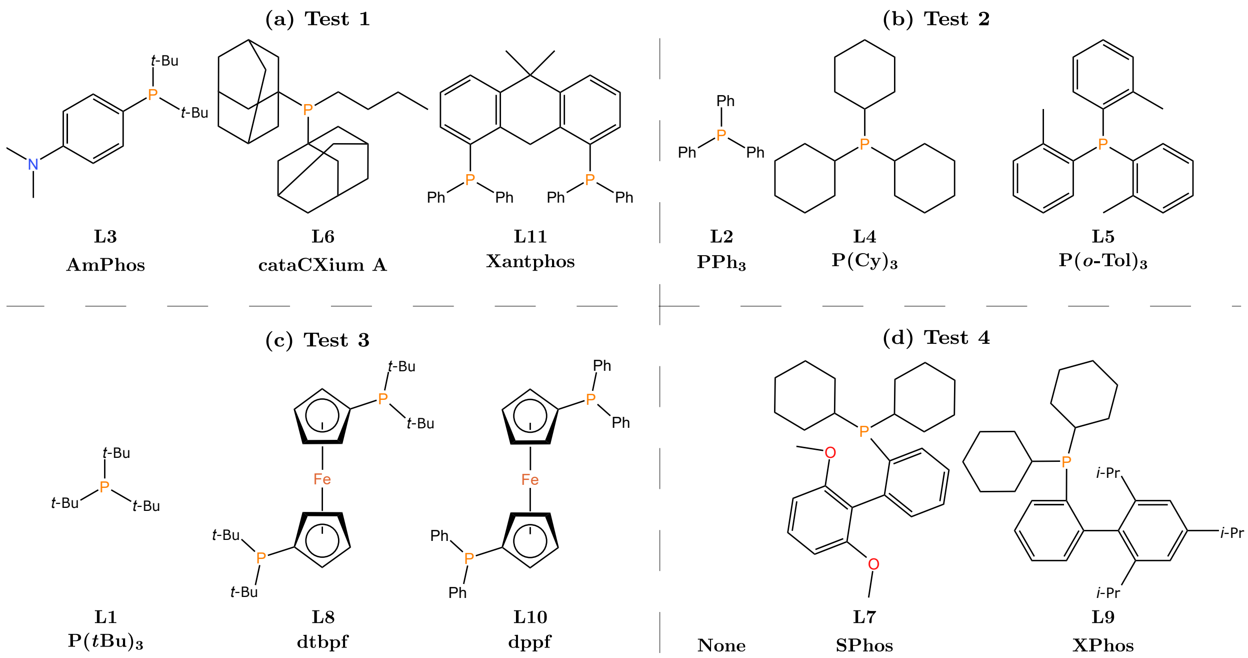

Figure S3: Ligands (L1-11, None) and the corresponding groups under out-of-sample conditions (Tests 1-4) of the Suzuki-Miyaura dataset.

4 Results of machine learning predictions

4.1 Results of ablation study for the multi-view learning

We performed five trials of yield prediction using ReaMVP to investigate the impact of the data view for the predictor. Table S2 presents the detailed prediction results.

Table S2: Performance (R^2) of the Buchwald-Hartwig dataset under out-of-sample conditions with different views. Bold entries highlight the best performance.

Split type	1D	3D	1D+3D	2D+3D
Test 1	**0.854 ± 0.013**	0.827 ± 0.033	0.844 ± 0.004	0.817 ± 0.018
Test 2	0.885 ± 0.016	0.865 ± 0.015	**0.896 ± 0.004**	0.858 ± 0.024
Test 3	0.771 ± 0.010	0.741 ± 0.100	**0.792 ± 0.025**	0.638 ± 0.030
Test 4	0.680 ± 0.035	0.611 ± 0.081	**0.693 ± 0.038**	0.636 ± 0.021
Plate 1	0.757 ± 0.017	0.745 ± 0.048	**0.785 ± 0.011**	0.717 ± 0.012
Plate 2	0.207 ± 0.111	0.171 ± 0.089	0.349 ± 0.129	**0.414 ± 0.039**
Plate 3	**0.798 ± 0.013**	0.733 ± 0.127	0.779 ± 0.017	0.730 ± 0.021
Plate 2 new	0.619 ± 0.029	0.618 ± 0.032	**0.689 ± 0.026**	0.626 ± 0.006

4.2 Results of ablation study for the pre-training stages

We performed five trials of yield prediction using ReaMVP to investigate the impact of the pre-training stages. Table S3 presents the detailed prediction results.

Table S3: Performance (R^2) of the Buchwald-Hartwig dataset under out-of-sample conditions with different pre-training strategies. Bold entries highlight the best performance.

Split type	stage I only	stage II only	both
Test 1	0.810 ± 0.007	0.828 ± 0.014	$\mathbf{0.844 \pm 0.004}$
Test 2	0.878 ± 0.008	0.887 ± 0.023	$\mathbf{0.896 \pm 0.004}$
Test 3	0.729 ± 0.016	0.763 ± 0.008	$\mathbf{0.792 \pm 0.025}$
Test 4	0.619 ± 0.048	0.673 ± 0.037	$\mathbf{0.693 \pm 0.038}$
Plate 1	0.765 ± 0.021	0.764 ± 0.022	$\mathbf{0.785 \pm 0.011}$
Plate 2	$\mathbf{0.401 \pm 0.032}$	0.312 ± 0.083	0.349 ± 0.129
Plate 3	0.770 ± 0.023	$\mathbf{0.782 \pm 0.015}$	0.779 ± 0.017
Plate 2 new	0.688 ± 0.010	$\mathbf{0.691 \pm 0.021}$	0.689 ± 0.026

4.3 Results of ablation study on Align and Contrast operations for the self-supervised pre-training

We performed five trials of yield prediction using ReaMVP to investigate the impact of Align and Contrast operations. Table S4 presents the detailed prediction results.

Table S4: Performance (R^2) of the Buchwald-Hartwig dataset under out-of-sample conditions with varying weighting coefficients. Bold entries highlight the best performance.

Split type	$\lambda = 0.1$	$\lambda = 0.5$	$\lambda = 1$
Test 1	0.830 ± 0.017	$\mathbf{0.844 \pm 0.014}$	$\mathbf{0.844 \pm 0.004}$
Test 2	0.888 ± 0.012	0.888 ± 0.008	$\mathbf{0.896 \pm 0.004}$
Test 3	0.739 ± 0.016	0.754 ± 0.029	$\mathbf{0.792 \pm 0.025}$
Test 4	0.672 ± 0.042	0.651 ± 0.029	$\mathbf{0.693 \pm 0.038}$
Plate 1	0.766 ± 0.010	0.776 ± 0.017	$\mathbf{0.785 \pm 0.011}$
Plate 2	0.240 ± 0.057	0.208 ± 0.127	$\mathbf{0.349 \pm 0.120}$
Plate 3	0.750 ± 0.017	0.771 ± 0.010	$\mathbf{0.779 \pm 0.017}$
Plate 2 new	0.622 ± 0.033	0.596 ± 0.021	$\mathbf{0.689 \pm 0.026}$

4.4 Results of non-deep-learning methods

We performed five trials of yield prediction using non-deep-learning methods to offer a better understanding of comparisons between different methods. Reaction features are provided by Mandana Saebi et al. [12]. We employed an extensive grid search in five cross-validations to choose the best hyper-parameters. Table S5 presents the detailed prediction results.

Table S5: Performance (R^2) of the Buchwald-Hartwig dataset under out-of-sample conditions. [a]With RDKit features. [b]Without RDKit features. The bold entry is better than that of deep-learning methods.

Split type	RF[a]	RF[b]	SVM[a]
Test 1	0.770 ± 0.002	0.810 ± 0.002	0.698 ± 0.0
Test 2	0.729 ± 0.003	0.716 ± 0.003	0.746 ± 0.0
Test 3	0.632 ± 0.002	0.593 ± 0.002	0.564 ± 0.0
Test 4	0.534 ± 0.004	0.568 ± 0.005	0.284 ± 0.0
Plate 1	0.675 ± 0.000	0.682 ± 0.002	0.562 ± 0.0
Plate 2	0.110 ± 0.001	0.139 ± 0.001	-0.040 ± 0.0
Plate 3	0.765 ± 0.003	$\mathbf{0.790 \pm 0.001}$	0.462 ± 0.0
Plate 2 new	0.626 ± 0.003	0.613 ± 0.004	0.474 ± 0.0
Halide Br	-0.100 ± 0.010	-0.443 ± 0.012	0.212 ± 0.0
Halide Cl	-1.060 ± 0.020	-2.092 ± 0.014	-0.902 ± 0.0
Halide I	0.553 ± 0.066	0.592 ± 0.002	-1.013 ± 0.001
pyridyl	-0.138 ± 0.008	-0.497 ± 0.057	-0.580 ± 0.001
nonpyridyl	0.160 ± 0.030	-0.494 ± 0.003	-0.584 ± 0.0

4.5 Results of the data from electronic laboratory notebooks

There are 750 reactions in the ELN Buchwald-Hartwig dataset [12]. We perform the same ten random splits provided by the original paper. Table S6 presents the detailed prediction results. The results of RF, SVM, ContextPred, EdgePred, AttrMasking, and W/O pre-training are directly from the original paper [12].

Table S6: Performance of the ELN Buchwald-Hartwig dataset. [a] With RDKit features. [b] Without RDKit features. Bold entries highlight the best performance.

Method	MAE	RMSE	R^2
RF[a]	**20.320 ± 0.769**	**25.270 ± 0.937**	**0.275 ± 0.040**
RF[b]	20.560 ± 0.728	25.480 ± 0.882	0.264 ± 0.032
SVM[a]	20.900 ± 0.800	-	0.222 ± 0.057
ContextPred	22.0 ± 0.2	-	0.177 ± 0.014
EdgePred	23.1 ± 0.2	-	0.129 ± 0.011
AttrMasking	22.2 ± 0.2	-	0.143 ± 0.008
W/O pre-training	22.0 ± 1.1	-	0.132 ± 0.045
YieldBERT	22.589 ± 2.304	27.468 ± 2.005	0.143 ± 0.102
YieldBERT-DA	21.581 ± 2.192	26.973 ± 1.981	0.171 ± 0.112
UA-GNN	20.635 ± 1.127	26.499 ± 1.027	0.203 ± 0.054
ReaMVP	20.692 ± 1.330	26.364 ± 1.289	0.212 ± 0.057

5 Initial features of atoms in the SchNet model

We encode the features of an atom into vectors and then concatenate them. Table S7 presents the initial features in a total of 133 dimensions. It is mapped into 128-D space by an embedding layer before being input into the SchNet [34] model.

Table S7: Initial features of atoms.

feature	description	size
atom type	type of the atom (e.g., C, N, O)	101
degree	number of connected bonds	7
formal charge	electrical charge	6
chiral tag	chiral information	5
Hs	number of bonded hydrogens	6
hybridization	e.g., sp, sp^2, sp^3	6
aromaticity	whether the atom is in an aromatic system	1
mass	0.01 times the mass of the atom	1

6 Yield distribution of the pre-training dataset

Figure S4 presents the yield distributions in USPTO-2 and USPTO-CJHIF by adding more reactions with low yields from CJHIF [23]. The percentage of chemical reactions with yields

below 50% increases from 29% to 41%.

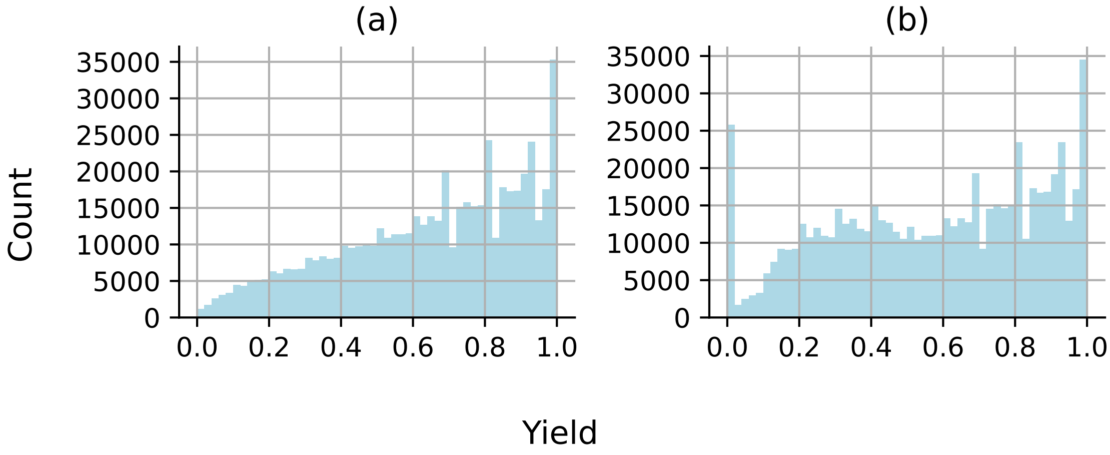

Figure S4: The yield distribution in USPTO-2 (a) and USPTO-CJHIF (b).
